# Animal Tuberculosis

**Published:** 1880-07

**Authors:** Allen S. Heath

**Affiliations:** Professor of Cattle Practice, Columbia Veterinary College; President Farmers’ Club, Etc.; New York


### ART. XII.—ANIMAL TUBERCULOSIS (CONSUMPTION).

### ITS TRANSMISSABILITY TO THE HUMAN SUBJECT THROUGH DISEASED MEAT AND MILK.

BY ALLEN S. HEATH, M.D.,

PROFESSOR OF CATTLE PRACTICE, COLUMBIA VETERINARY COLLEGE; PRESIDENT FARMERS' CLUB, ETC.

EVERYTHING which appears to affect the health of mankind has been alleged to be a cause of pulmonary consumption; such as deficiency and poor quality of food, lack of pure air, light, warmth and exercise; in fact, everything which impairs the nourishment of the body, induces poverty of the blood, or depresses the nervous system.

These or similar kinds of influence affect our milk cows quickly and profoundly, and, as a matter of course, the quality of the milk suffers to a greater or less extent. The excessive production of milk is an artificial condition to which the system of our bovines has been brought by long and careful breeding, feeding and management, and they therefore are more susceptible to the baneful influences than natural and unstimulated animals are under proper normal conditions.

Tuberculosis prevails extensively among domestic animals all over the entire globe, and especially in populous and overcrowded localities, where, from necessity, defective food, lack of care, filth, bad air, defective ventilation, want of sun-light, and want of exercise, all add accumulating conditions favorable for the development of this fatal disease. In Mexico 34 per cent. of all slaughtered bovines supply tuberculous meat; and it is probable that milk cows under similar circumstances are affected to the fearful extent of 50 per cent.

Van Hertsen, of Belgium, found tubercles in all the tissues of an apparently healthy bull seven years old. It is astonishing how extensive even in pleuro-pneumonia may be the lesions of the disease in both lungs, and yet the animals seem to be in fair condition, keep good appetites, and consume the usual allowance of food. Temperament, as in man, must account for much of this anomaly.

Cows confined in dark, damp, unventilated city stables become tuberculous to the extent of seventy-five per cent. in the course of time. Speaking of this subject, Fleming says: "It must be borne in mind that there are few animals which have been kept for any length of time in cow-sheds, and fed and milked in the usual manner, which are not more or less phthisical; more part cularly is this the case if the dwellings are bad."

Again: The milk of tuberculous cows is of poor quality, being deficient both in fat and albuminoids, the essential elements of healthy food, to say nothing of its liability to become the exciting cause of disease.

That the milk and meat of consumptive animals produce fatal diseases very like consumption in persons and animals consuming them, is a fact not to be successfully controverted, and compels the physician and sanitarian to warn the public against diseased meat and diseased milk, and also indicates to the boards of health a *plain* duty in preventing the use of all kinds of diseased or unsound food.

Vogel says that "no cow's milk should be given to children, unless it is first rendered alkaline by adding a teaspoonful of soda solution (a drachm to six ounces of water) to every meal, to to prevent intestinal catarrh."

The food supply of great cities is of the chiefest importance to the health of their citizens; and where the infant mortality is great, the milk supply should be guarded with the greatest assiduity.

In 1865, Villemin proved by repeated experiments that it was possible to produce consumption in previously healthy animals. He found that finely-divided tuberculous matter, when introduced under the skin of rabbits and guinea-pigs, produced pulmonary tuberculosis in three weeks—thus proving from these experiments that tuberculosis should be classed as a specific disease, capable of being contracted by inoculation, like small-pox or syphilis. Numerous pathologists have verified Villemin's experiments.

Dr. Wilson Fox and Dr. Sanderson found that pneumonic matter, pus, putrid matter, etc., would produce disease in healthy animals, and transmit it through their meat and milk to dogs, cats, hogs, and through milk to young children and animals to which it had been fed.

Gerlach in numerous experiments demonstrated that the milk of tuberculous cattle will produce consumption in creatures fed on it, and many other pathologists have verified his experiments with similar results. But it is not our purpose to go into detail in these matters, but the rather to be content with proven facts and their application.

Kleibs has produced tubercles in rabbits, guinea-pigs, and dogs, by giving them the milk of diseased cows. This kind of milk given to young children, and particularly if given during dentition and the excessive heat of mid-summer, which, in unfavorable localities for proper ventilation, often reaches from 90° to 100° Fahr., excites irritation of the stomach and bowels, resulting in chronic catarrhs of these membranes, which render them keenly susceptible to the poisonous influences of tuberculous matter long before the tubercle finds lodgment in the lungs. It is not often that the intestinal mucous membranes of young children, who die from what is supposed to have been *cholera infantum*, are examined after death; but, doubtless, tubercles in these tissues would frequently be found in many cases in which their presence was not even suspected, particularly as the other affection is so common and fatal in early infancy.

There is little doubt that diseased milk and diseased meat irritate the mucous membranes of the stomach and bowels, and, doubtless, many cases of so-called *cholera infantum* are the result of tuberculosis of these tissues. This inference is drawn from Zeimssen, as quoted from Ruehle. He says: "Whether ulcerative processes are directly excited by this means, and consumption is produced by infection from the discharges, is uncertain; but there is no doubt that, in individuals who are predisposed to consumption, the intestinal diseases may continue for a long time before we can discover anything abnormal in the lungs, and that, after death from phthisis, in such cases the intestines exhibit tuberculous ulceration."

At the American Medical Association, at its thirty-first annual meeting, held in New York, June 1st, 1880, J. S. Lynch, M.D., Chairman of the Section of the Practice of Medicine, etc., read a most valuable paper, in which he referred the fearful mortality of young children to unsanitary conditions, and dwelt upon the great influence of unwholesome and diseased food as a marked cause of death.

Ruehle regards Villemin's experiments, in reproducing the disease by inoculation of animals with tuberculous matter, as most valuable, and as a complete victory over Laennec's theory.

Jacobi reports that a dog which ate the sputa of his consumptive master died of consumption.

Fleming says: "It is certain that tuberculosis is a somewhat common and very destructive disease among dairy cattle especially, and more especially those of towns." And consumption is one of the most fatal diseases of large cities, and doubtless, to a considerable degree dependent upon this diseased milk.

Marasmus is undoubtedly more or less attributable to the same cause. Infant mortality is frightful in the excessively hot weather of parts of July and August when many causes add to the irritability of the digestive apparatus, rendering the poisonous influences of acid, impure, or tuberculous milk more effective in the production of fatal diseases. In just these debilitating days of heat do cows also suffer most, and the result must be to supply worse milk.

Niemeyer says "that the predisposition to consumption is strongest in persons of feeble and delicate constitution, and is especially so in children poorly nourished. The children fed on the milk of tuberculous cows must, of necessity, suffer in a two-fold sense—from bad food and poisonous food also."

In the production of consumption in city children, and especially in the tenement house population, these accumulated and concentrated influences are more potential as the quality of the milk and other food is inferior and often deficient in quantity. Cheap food and cheap milk are usually purchased by those who, above all others, suffer most from unsanitary conditions; and generally cheap food and cheap milk are both bad and dangerous especially from tuberculous animals.

From these facts it is apparent that there is great danger in eating uncooked beef, for fear of contracting consumption, if, indeed, cooking does in fact destroy its virus. The sources from which consumption are derived are now known to be infinitely more numerous than the older pathologists suspected.

It is more dangerous to eat the milk of consumptive animals than to eat the meat; for the milk is seldom boiled, while the meat is always cooked. Cooking is a most valuable sanitary measure in vegetable as well as animal articles of food.

Cows living under bad hygienic conditions, as in man, under similar conditions, are predisposed to tuberculosis in themselves and thus their milk becomes poisonous to children who were previously debilitated by unsanitary conditions, such as are almost inseparable from residence in tenement houses. In the suburbs of the cities of New York and Brooklyn, most of the cows are diseased from this cause and by being fed on unsound food.

In a future paper I shall pay my special respects to city cows and cow-stables, and I shall hope to show, I trust, that the milk from diseased cows poisons thousands of children, who are supposed to die from *cholera infantum*, when in fact they die from tubercles of the intestines, which produce irritation sufficient to cause a rapidly wasting diarrhœa. Consumption is infinitely more common in city-kept cows than it is believed to be even by physicians. I recently called Prof. Chandler's attention to the unusual number of cows crowded into city stables, and, from the fact of his taking copies of notes sent me by James D. Hopkins, D. V. S., of the State Commission for the eradication of pleuro-pneumonia, I feel assured that so efficient a sanitarian as he is will have these shameful nuisances promptly abated, and future dangers to health prevented.

It has been my aim to be suggestive in this paper rather than ustive.

A similar paper was read before the Farmers' Club, since which I have had many communications of inquiry, and several complimentary notices in reference to the timely and important character of the subject in a sanitary point of view. I shall feel much gratification in having the subject further illuminated and discussed by medical men and sanitarians.

In closing this article I wish to state that since the foregoing was written, I find that in 1874 Fleming wrote and published a paper on Tuberculosis in Cattle, which I regret not to have seen, and to which he refers in an article on the same subject in his *Veterinary Journal* of May, 1880. In this last paper I now see that he refers to the possibility that infantile diarrhœa may to some extent be referred to tuberculous milk. This exactly agrees with what I wrote a month previous to the publication of his article, and which gives me great pleasure to learn, since he could not have then seen my paper.

Breeding from tuberculous cows perpetuates the disease by heredity.

Herr Adonis very naturally and correctly regards cows as more subject to the disease than other cattle; and Zippillius is of opinion that bovine tuberculosis is more common than human tuberculosis.

